# The role of CDPKs in plant development, nutrient and stress signaling

**DOI:** 10.3389/fgene.2022.996203

**Published:** 2022-09-30

**Authors:** Simon Dontoro Dekomah, Zhenzhen Bi, Richard Dormatey, Yihao Wang, Fasih Ullah Haider, Chao Sun, Panfeng Yao, Jiangping Bai

**Affiliations:** ^1^ Gansu Provincial Key Laboratory of Aridland Crop Science, Lanzhou, China; ^2^ College of Agronomy, Gansu Agricultural University, Lanzhou, China; ^3^ College of Resources and Environmental Sciences, Gansu Agricultural University, Lanzhou, China

**Keywords:** calcium, calcium-dependent protein kinases, stress tolerance, plants, biotic and abiotic stress

## Abstract

The second messenger calcium (Ca^2+^) is a ubiquitous intracellular signaling molecule found in eukaryotic cells. In plants, the multigene family of calcium-dependent protein kinases (CDPKs) plays an important role in regulating plant growth, development, and stress tolerance. CDPKs sense changes in intracellular Ca^2+^ concentration and translate them into phosphorylation events that initiate downstream signaling processes. Several functional and expression studies on different CDPKs and their encoding genes have confirmed their multifunctional role in stress. Here, we provide an overview of the signal transduction mechanisms and functional roles of CDPKs. This review includes details on the regulation of secondary metabolites, nutrient uptake, regulation of flower development, hormonal regulation, and biotic and abiotic stress responses.

## 1 Introduction

Calcium is an important messenger in plants that coordinates a wide range of physiological processes in response to external and endogenous factors. A transient increase in cytosolic calcium often occurs during plant stress responses (Mahajan et al., 2008) and produces specific calcium signatures that can be detected by various calcium sensors to trigger downstream effects. These effects are manifested in changes in protein phosphorylation and gene expression patterns (Kudla et al., 2010). Ca^2+^-dependent protein kinases (CDPKs), calmodulin (CaM), calmodulin-like proteins (CMLs), calcineurin B-like proteins (CBLs), and their interacting kinases (CIPKs) are four major classes of calcium-sensing proteins in plants (Hashimoto and Kudla 2011; Batistic and Kudla 2012). CDPKs are Ca^2+^-regulated serine/threonine protein kinases encoded by a large family of multigene found in plants, green algae, protists, and oomycetes (Valmonte et al., 2014). Among calcium sensors, CDPKs are the only ones that combine sensing activity *via* EF-hand calcium-binding motifs with response activity *via* the protein kinase domain in a single protein (Yip Delormel and Boudsocq, 2019).

CDPKs consists of four characteristic domains including a variable N-terminal domain containing myristoylation and palmitoylation sites, a catalytic ser/thr protein kinase domain that phosphorylates the serine and threonine residues of its substrates, an autoinhibitory domain acting as a pseudosubstrate blocking enzyme activity in the absence of Ca^2+^ stimulation, and a C-terminal regulatory calmodulin-like domain with one to four conserved EF-hand motifs for calcium-binding (Cheng et al., 2002; Liese and Romeis, 2013; Valmonte et al., 2014; Xu et al., 2015). CDPKs recognize Ca^2+^ signals *via* the EF-hand motif, a distinctive and conserved helix-loop-helix structure composed of 12 amino acids that confer the Ca^2+^-binding activity. Because EF-hands typically occur in pairs as a discrete domain, the majority of CDPK family members have two, four, or six EF-hands (Shi et al., 2018; Yip Delormel and Boudsocq, 2019). In certain conditions, the pairing exhibits positive interaction, reducing the Ca^2+^ signal required to achieve protein saturation. Ca^2+^ binding to the CDPK cause a structural change that promotes the CDPK’s interaction with its target proteins or alters the CDPKs enzyme activity. CDPKs are one general class of EF-hand-containing proteins found in plants that translate chemical signals into diverse biochemical responses (Shi et al., 2018; Yip Delormel and Boudsocq, 2019). Many CDPK genes have been identified in plants, 34 in *Arabidopsis thaliana* (Cheng et al., 2002), 31 in rice (*Oryza sativa*) (Ray et al., 2007), 29 in tomato (*Solanum lycopersicum*) (Wang et al., 2016), 18 in melon (*Cucumis melo*) (Zhang et al., 2017), 31 and 32 in *Cucurbita maxima* and *C. pepo*, respectively (Wei et al., 2019), and 19 in cucumber (*Cucumis sativus*) (Xu et al., 2015).

CDPKs have been found in various subcellular locations, such as the plasma membrane, cytosol, nucleus, endoplasmic reticulum, peroxisomes, outer mitochondrial membrane, and oil bodies, implying that they play roles in a variety of physiological processes (Dammann et al., 2003; Harper et al., 2004). They are involved in starch and protein accumulation in immature rice seeds, tolerance to cold, salt, drought stress, and abscisic acid (ABA) response in *Arabidopsis*, rice, and watermelon (*Citrullus lanatus*) (Mehlmer et al., 2010; Asano et al., 2011; Asano et al., 2012b; Wei et al., 2019), defense response in tobacco (*Nicotiana tabacum*) (Romeis et al., 2000) and tomato (Chico et al., 2002), nodulation in *Medicago truncatula* (Gargantini et al., 2006), and pollen tube growth in petunia and abscisic acid (ABA) response in *Arabidopsis* (Nakata et al., 2009). CDPKs regulate seed germination (Jiang et al., 2013), pigment accumulation, and fruit development (Pawełek et al., 2015), and promote grain filling in rice which shortens the crop duration (Manimaran et al., 2015).

Plants are constantly faced with environmental stresses, and adequate stress management requires a high energy expenditure and often results in slowed or completely impaired growth. Therefore, it is in the best interest of plants to develop mechanisms to prioritize simultaneous external challenges to balance growth and stress responses (Seybold et al., 2019) so that plants can escape, avoid, or tolerate environmental changes (Blum, 1996; Lerner, 2018). Several mechanisms come into play, depending largely on the type, severity, and duration of stress, and may also vary among plant species and developmental stages (Pinheiro and Chaves, 2011). Among the known mechanisms, pathways involving CDPKs are one of the best-studied cases. CDPKs are promising candidates for a role in coordinating the trade-off between stress tolerance and growth (Claeys and Inzé, 2013). Therefore, this review will focus on the progress of CDPK research, particularly on how CDPKs regulate plant stress tolerance and promote growth and development.

## 2 Evolutionary pattern and functional changes in the CDPK family in plants

The CDPK gene family has a long evolutionary history that can be traced back to the earliest land plants, such as pteridophytes (*Selaginella moellendorffii*) and bryophytes (*Physcomitrella patens*), both of which have at least 35 CDPKs in their genomes (Hamel et al., 2014). CDPK homologs were previously thought to be plant-specific, but they have now been discovered in ciliates and apicomplexan parasites (Zhang and Choi. 2001; Billker et al., 2009). The first CDPK gene appeared prior to the basal split between green plants and alveolate protists (Zhang and Choi. 2001). The cucumber’s genome encodes 19 *CsCDPKs* and 18 *CmCDPKs* in melon, compared to 34 *AtCPKs* in *Arabidopsis* (Harper and Harmon 2005; Xu et al., 2015; Zhang et al., 2017), 25 in potato (Bi et al., 2021), 30 *PtCDPKs* in Poplar (Zuo et al., 2013), and 31 *OsCDPKs* in rice (Ray et al., 2007). The lower number of CDPK genes in some plants e.g. cucumber and melon may be attributed to a lack of whole genome duplication (WGD) events following the gamma WGD, as seen in *Arabidopsis*, or the WGD after the split between eudicots and monocots, as seen in rice (Jiao et al., 2011). Another possible explanation could be the cucumber or melon genome’s low number of segmental duplications and tandem gene duplications (Asano et al., 2012a). However, recent duplication events in *Arabidopsis, Populus*, and rice may have resulted in an increase in the number of CDPK family members in their genomes (Remington et al., 2004).

The evolutionary history of CDPKs phylogenetic groupings and gene structure can be used to predict the functional specificity between paralogs. The *Arabidopsis* genome encodes 34 CDPKs, grouped into four distinct clusters (Boudsocq and Sheen, 2013) which is similar to several angiosperms CDPK family’s basal architecture such as tomato (Hu et al., 2016), Chinese cabbage (Wu et al., 2017), potato (Dekomah et al., 2022), maize (Li et al., 2018). However, recent studies in sweet potato (*Ipomoea spp*.) revealed more than four clusters, with three CDPKs forming a fifth cluster (V) (*IbCDPK35, ItfCDPK35, ItbCDPK35*) that was speculated to have resulted from chromosomal hybridization (Li et al., 2022). With gene structure, it has been proven that when CDPK sequences within a genome belonged to the same evolutionary cluster they showed similar intron-exon patterns. Although this is not always the case, but highly homologous CDPKs have very similar intron-exon patterns. One example is the comparison of *AtCPK4* and *AtCPK11* to *AtCPK12*. In terms of protein sequence, these three sequences share a lot of similarities. *AtCPK4* and *AtCPK11* have very similar intron-exon patterns, but the *AtCPK12* gene structure differs from these two (Valmonte et al., 2014). In terms of function, *AtCPK4* and *AtCPK11* display functional similarity, but *AtCPK12* was different (Valmonte et al., 2014). CDPKs, CMLs, CaMs, and CBLs account for more than one-third of all EF-hand containing domain found in plant genomes (Mohanta et al., 2017). The overall trend in the evolutionary process of green plants is a significant and sustained increase in the number of EF-hand domains, whereas the number of EF-hands was initially very low in early algae, indicating that Ca^2+^ sensing appeared to experience differential expansion and functional specialization in CDPKs, CMLs, CaMs, and CBLs. Abiotic stresses and plant morphological complexity may also be linked to changes in the number of Ca^2+^-sensing genes and EF-hand motifs (Tong et al., 2021). Considering the evolutionary history of CDPKs it is established that, this multigene family is conserved among angiosperms based on their basal architecture and gene structure which has contributed to expanding their signaling roles.

## 3 Gene duplication

Plant phenotypes have been significantly altered during evolution to adapt to environmental changes by transforming the form and function of genes. Most land plants have undergone polyploidization, which has resulted in Whole-Genome Duplication (WGD) and allowed duplicated genes to diverge in function. Each of these genes went through one of three transformations: neofunctionalization, subfunctionalization, or non-functionalization (pseudogenization). These outcomes allowed duplicated genes to acquire functional diversification, culminating in more complex organisms (Wu et al., 2017; Rensing, 2014). Neofunctionalization is an adaptive process in which one copy of a duplicated gene mutates and ultimately performs a novel function (that the ancestral sequence cannot perform. This is one of the mechanisms that can result in the long-term retention of both copies of a gene after duplication. A classic example was found in the functional diversification of cucumber *CsCDPKs* paralogs gene pair (*CsCDPK1* and *CsCDPK3*) in cluster A. Both genes lost motif 9 whiles sharing the same intron phase, with one of the duplicates changing its expression pattern to be more tissue (fruit) specific indicating neofunctionalization and may play a significant role in fruit development (Xu et al., 2015). The duplicate gene pair in grapevine (*VvCPK5* and *VvCPK11*) in cluster C underwent neofunctionalization where *VvCPK5* evolved to become more specific in pollen development while *VvCPK11* retained its housekeeping role (Chen et al., 2013). Subfunctionalization is a related process in which duplicated gene copies diverge to perform specific functions than the ancestral gene, which can help to explain the duplicates’ short-term fate and can be considered as a transition state towards neofunctionalization (Rastogi and Liberles, 2005). In the case of *CsCDPK8* and *CsCDPK17* in cluster B, *CsCDPK17* is primarily expressed in tendrils, whereas *CsCDPK8* is expressed at a relatively similar level in all tissues maintaining their housekeeping role, suggesting subfunctionalization (Xu et al., 2015). For *VvCPK8* and *VvCPK9* in cluster A, both genes were found to express in fewer tissues such as pollens compared to *VvCPK13* in the same cluster A which retained its ancestral function in stress response (Chen et al., 2013).

Since it diverged from *Carica papaya*, the genome of *A. thaliana* has undergone a paleohexaploidy (β) duplication, as well as two subsequent genome duplications (α and γ), in addition to rapid DNA sequence divergence and extensive gene loss (Jiao et al., 2011). The evolutionary history of grapevine CDPKs occurred in the absence of the alpha (α) and beta (β) whole-genome duplications experienced by *A. thaliana* (Chen et al., 2013). The CDPK family in angiosperms is characterized by the presence of numerous paralogs with high levels of homology. These closely related genes most likely arose as a result of recent duplication events and, as a result, have not yet expanded significantly (Hamel et al., 2014). In some instances, duplication appears to predate the monocot-eudicot split (e.g., *OsCPK17/27, AtCPK1/2, and PtCPK1/2*), whereas, in others, duplication appears to be specific to a particular lineage (*OsCPK12* and *AtCPK29* versus *PtCPK29-1/29-2*) (Hamel et al., 2014). Some duplicated regions in CDPK-SnRK protein kinases found in *A. thaliana* indicated that CDPK-SnRK protein kinases are paralogs that emerged by divergence after genome duplication events (Hrabak et al., 2003). *Arabidopsis* and maize CPK genes have undergone both segmental and tandem duplication, which has contributed to the CPK family’s expansion. In *Populus*, segmental duplication was the major factor in the expansion of CPK genes (Zuo et al., 2013). A study of gene chromosomal distribution reveals that few CDPK genes occur as tandem duplicates within angiosperms. *CPK20-1/20-3* and *CPK20-2/20-4* in poplar (Zuo et al., 2013) are notable exceptions, as well as a cluster of five Group II CDPKs in *Arabidopsis* (*CPK21/22/23/27/31*) (Cheng et al., 2002; Hrabak et al., 2003). As identified for mitogen-activated protein kinases (MAPKs) (Hamel et al., 2014), the expansion of the CDPK family from angiosperms depended largely on large-scale DNA rearrangements, namely whole-genome or large segmental duplications. No tandem duplication of CPK genes has occurred in the rice genome (Asano et al., 2012a). Two duplicated gene pairs (T*kCPK26/TkCPK27; TkCPK27/TkCPK*) were found in *Taraxacum koksaghyz* indicating accelerated evolution rates (Zhu et al., 2010). Most ancestral CDPKs of plants had diverse functions in the development of reproductive structures and maintenance of other cellular processes; however, several duplication events over the years have contributed to expanding, gaining, or retention of CDPK functions in response to changing environmental conditions.

## 4 Structure and activation of CDPK

**FIGURE 1:**
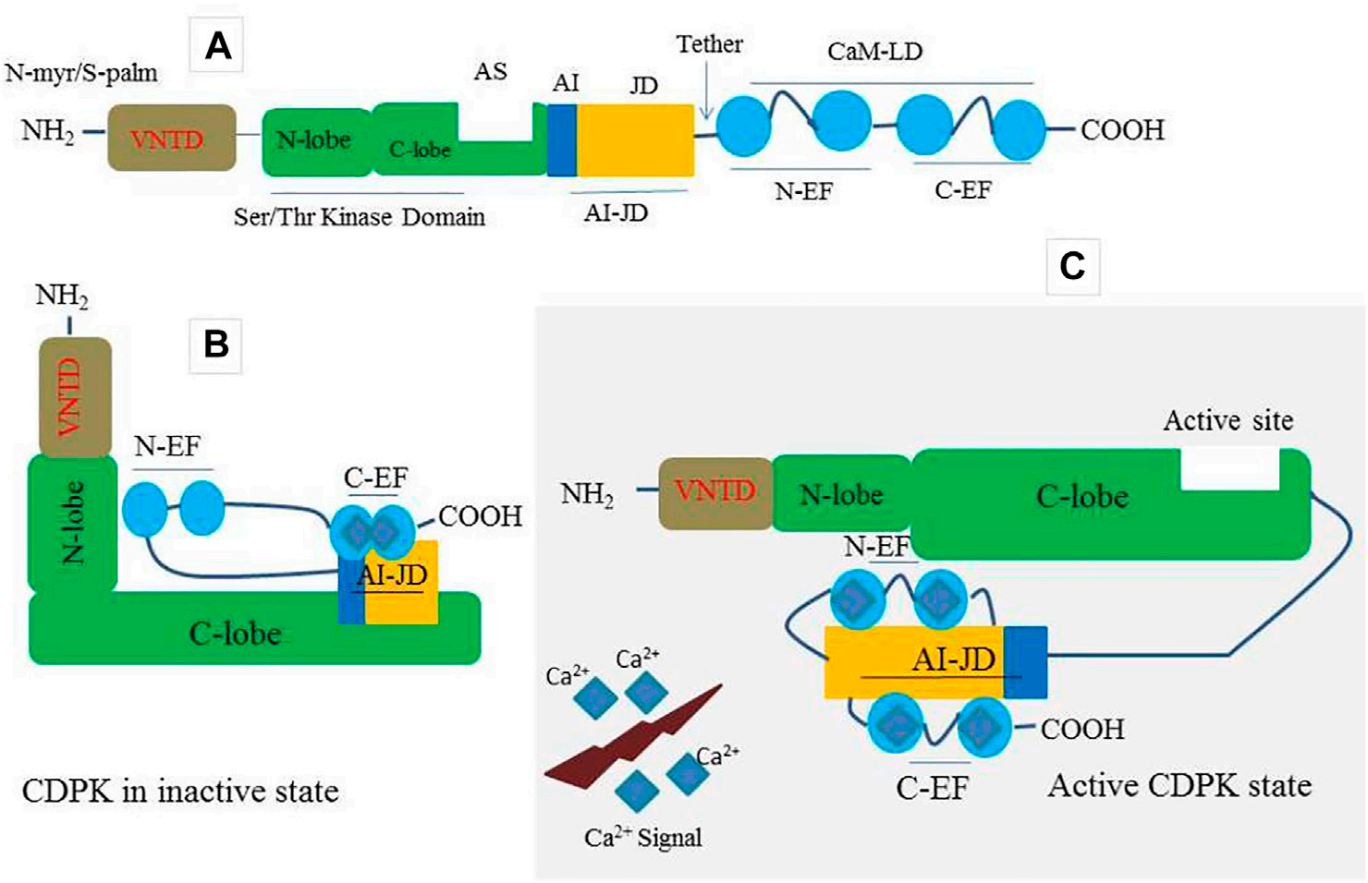
A prototype of CDPK protein structure and its decoding mechanism. **(A)** A typical CDPK comprises a variable N-terminal domain (VNTD) which contains (Myristoylation site and Palmitoylation site), serine/threonine kinase domain, active site (AS), auto-inhibitory junction domain (AI-JD) made up of [auto-inhibitor (AJ) and junction domain (JD)], and a C-terminal CaM-like domain (CaMLD) which harbor (four EF-hands) for Ca^2+^ binding. **(B)** CDPK in an inactive state with AI acting as a restraining molecule. In a resting position, the AI-JD interacts with the C-lobe and C-EF by binding in the AS whiles the N-lobe interacts with N-EF. **(C)** CDPK in an active state. Upon increase in cytosolic Ca^2+^ concentration, the AI-JD detaches from the AS as a result of large conformation changes that occurred in the kinases domain. Ca^2+^ ions bind with the C-terminal lobe (C-EF) and N-terminal lobe (N-EF) and then interact with AI-JD on both sides.

Plants have a large number of calcium-binding proteins that act as cellular Ca^2+^ sensors as well as the first point of information translation (McCormack et al., 2005; Kim et al., 2007; Kudla et al., 2010). As one of the Ca^2+^ sensors, CDPKs have four domains including; a calmodulin-like domain (CaM-LD) or calcium-binding domain, a serine/threonine protein kinase domain, a variable N-terminal domain (VNTD), and an autoinhibitory junction domain (AI-JD) (Batistic and Kudla 2012) (Figure 1A). At its C-terminus, the auto-inhibitory-junction domain contains a pseudosubstrate auto-inhibitor (20∼30 amino acids) and a junction region, whereas the CaM-LD domain contains four EF-hand Ca^2+^-binding motifs classified into two lobes, with the N-lobe having a lower calcium affinity than the C-lobe (Christodoulou et al., 2004; Liese and Romeis 2013; Yip Delormel and Boudsocq, 2019). The covalent tethering of the CaM-LD to its regulatory-junction region in CDPKs is a unique feature of the CaM superfamily (Chandran et al., 2006). In an inactive state, the auto-inhibitor binds to the adjacent kinase domain in the active site and prevents it from performing its activity (Figure 1B). When activated by increased Ca^2+^, Ca^2+^ ions loads into the EF-lobes, causing a structural change of CaM-LD. Meanwhile, the EF-lobes cling to the junction region, allowing the auto-inhibitor to be released. The kinase domain is then exposed and phosphorylated by an uncharacterized cellular kinase (Liese and Romeis 2013; Christodoulou et al., 2004; Klimecka and Muszy’nska, 2007) (Figure 1C). The activated CDPK can then recognize and phosphorylate its targets (Figure 1C).

CDPK proteins contain a variable N-terminal domain (VNTD) at the N- terminus (Hamel et al., 2014) (Figure 1A), which varies in amino acid sequence and length (Cheng et al., 2002). Although there is little information about the function of the VNTD, recent studies suggest that this region is involved in substrate specificity (Ito et al., 2010; Asai et al., 2013). Most flowering plants CDPK VNTDs also have predicted N-myristoylation and S-palmitoylation sites, which promote protein targeting to lipid membranes (Boudsocq and Sheen 2013; Zuo et al., 2013; Hamel et al., 2014; Ma et al., 2015) (Figure 1A). The membrane-anchoring of a CDPK was first demonstrated experimentally in rice (Mart’n and Busconi 2000); however, this phenomenon has since been demonstrated for many *Arabidopsis* candidates, as well as CDPKs from other flowering plant species (Boudsocq and Sheen 2013; Liese and Romeis 2013). Importantly, CDPKs that are membrane-targeted can also move away from membranes in response to stress signals (Gao et al., 2013). This feature most likely allows CDPKs to shuttle between various subcellular compartments in order to perform a broader range of cellular functions.

## 5 Functional characterization of the CDPK family in plants

### 5.1 CDPKs in secondary metabolite production

CDPKs have been linked to the regulation of secondary metabolite production through phosphorylation of the enzyme PAL (phenylalanine ammoniumlyase) (Zhang and Liu, 2015). *AtCPK1* could phosphorylate PAL *in vitro*, the first-step enzyme in Phe-derived phytoalexin biosynthesis. Overexpression of *AtCPK1* in soybean cells causes the accumulation of phytoalexins, the antimicrobial compounds, indicating the involvement of *AtCPK1* in defense (Cheng et al., 2001; Veremeichik et al., 2019). In addition, heterologous expression of *AtCPK1* in *Rubia cordifolia* cells was found to have a strong stimulatory effect on anthraquinone (AQ) production (Shkryl et al., 2011). Furthermore, by phosphorylating WRKY33, the pathogen-responsive CPK5 and CPK6 in *Arabidopsis* regulate camalexin biosynthesis (Zhou et al., 2020). It was shown that phosphorylation of WRKY33 by MPK3/6 promotes its transactivation, whereas CPK5/CPK6 phosphorylation increases its DNA binding ability (Zhou et al., 2020), implying that CPKs and MAPKs may function synergistically (Yang et al., 2020; Zhou et al., 2020). Aleynova-Shumakova et al. (2014) found that overexpression of *VaCPK20* or *VaCPK29* increased resveratrol production in *Vitis amurensis* cells whiles some CDPKs such as *HbCPK9/15* are associated with latex biosynthesis (Zhu et al., 2010; Xiao et al., 2017). On the other hand, *FaCDPK4* and *FaCDPK11* have been linked to drought stress tolerance where ABA is used as a signaling molecule to increase phenolics, anthocyanins, ascorbic acid, and sugar compounds (Crizel et al., 2020).

### 5.2 CDPKs regulate nutrient transport in plants

It is well understood that nitrate (NO_3_^−^) availability causes extensive transcriptional changes, which are mediated in part by changes in [Ca^2+^] in various cellular compartments. NO_3_^−^ induces distinct Ca^2+^ signatures, culminating in the activation of CDPKs and CIPKs, which phosphorylate channels, metabolic enzymes, and transcription factors (Liu et al., 2020). *AtCPK10/AtCPK30/AtCPK32*, for example, phosphorylates NIN-LIKE PROTEIN (NLP) transcription factors to modulate transcriptional responses to nitrate availability. An *Arabidopsis* triple-mutant of *cpk10 cpk30 cpk32* 3-day-old seedlings placed on a 3MBiP medium in the presence of 5 mM KNO_3_ for 5-8 days exhibits down-regulation of nitrate-responsive genes as well as impaired cotyledon greening and expansion (Liu et al., 2017). The triple-mutant-specific deficiency of cotyledon and leaf expansion in response to nitrate may be partly correlated with decreased expression of *CYP735A2 for trans-zeatin* synthesis (Kiba et al., 2013; Liu et al., 2017).

One of the most important macronutrients in crop production is nitrogen. *A. thaliana* roots gain access to ammonium (NH_4_^+^) by promoting the expression of ammonium transporters (AMTs) of the Ammonium Transporter/methylammonium permease/rhesus (AMT/MEP/Rh) protein superfamily (Loqué and von Wirén, 2004). AMTs are potent transporters with Km values in the micromolar (μM) range, exhibiting high substrate specificity for NH_4_^+^ and its methylated analog, methylammonium (MeA) (Loqué et al., 2006; Pantoja, 2012). Six AMTs have been identified in *Arabidopsis*. Of these AMT1; 1, AMT1; 2, and AMT1; 3, are the major transporters for high-affinity ammonium uptake into roots, with ammonium substrate affinities of 50, 234, and 61 μM respectively (Loqué et al., 2006; Yuan et al., 2007). AMT1; 1 and AMT1; 3 localized predominantly in the rhizodermal and cortical cells, form homo- and hetero-oligomers at the plasma membrane, and contribute 30–35% of the high-affinity ammonium uptake capacity (Yuan et al., 2007; Yuan et al., 2013). The function of AMT1:1 depends on CPK32-mediated phosphorylation at the non-conserved serine residue S450 in its C-terminal domain (Qin et al., 2020). The phosphorylated variant AMT1; 1^S450E^ but not the non-phosphorylatable variant AMT1; 1^S450A^ was found to wholly complement MeA insensitivity and restore increased ^15^NH_4_^+^ uptake in both *amt1;1* and *cpk32* mutants in transgenic plants (Qin et al., 2020). This suggests that CDPK positively regulates the uptake of both nitrate and ammonium in plant roots.

### 5.3 CDPKs in plant development

Pollen tube growth, which involves ions (Ca^2+^, H^+^, K^+^, and Cl^−^) and water fluxes regulated by a calcium gradient at the tube tip, is essential for successful fertilization. Some *Arabidopsis* CPKs are only expressed in pollen tubes, revealing major roles for CDPKs in this process (Gutermuth et al., 2013; Zhao et al., 2013). The CPK11-CPK24-SPIK pathway has also been identified in pollen tube cells (Zhao et al., 2013; Shi et al., 2018). Using patch-clamp analysis, it was discovered that an increased cytoplasmic Ca^2+^ concentration inhibited K^+^ influx in pollen tube protoplasts. Among 16 examined pollen-tube-expressed CDPKs, six *AtCPK* mutants differed significantly from wild-type plants in pollen germination or pollen tube growth (Zhao et al., 2013). The measurement of the K^+^ influx currents in *cpk11* and *cpk24* mutants revealed that both CPK11 and CPK24 are required for Ca^2+^-dependent inhibition of K^+^ influx channels and showed that these two CPKs function in the same pathway (Zhao et al., 2013). CPK11 can bind to and phosphorylate CPK24 *in vivo* (Shi et al., 2018). Further electrophysiological experiments revealed that the shaker pollen K^+^ influx channel (SPIK) may act as the substrate protein in this pathway, implying that CDPK can regulate pollen tube elongation through K^+^ influx regulation (Zhao et al., 2013).

CDPKs have been implicated in mediating Ca^2+^-regulated pollen tube growth as an important node in Ca^2+^ signaling pathways (Estruch et al., 1994; Moutinho et al., 1998). The influence of CPK in promoting pollen tube germination and growth was first found in maize (*Zea mays*), where inhibition of a pollen-specific CPK hindered both germination and polar growth (Estruch et al., 1994). Moreover, *PiCDPK1* and *PiCDPK2* have been shown to regulate pollen tube growth polarity and extension, respectively (Yoon et al., 2006). Genetic evidence has proven that CPKs are involved in pollen tube growth, *AtCPK17* and *AtCPK34* essentially help in maintaining pollen tube tip growth rate and facilitating response to tropism signals (Myers et al., 2009). Further research discovered two pollen-specific water and nonionic channels, NIP4; 1 and NIP4; 2, as substrates of *AtCPK34* that regulate pollen germination and tube growth (DiGiorgio et al., 2016). A negative gradient of anions at the pollen tube tip, which has an inverse correlation with Ca^2+^ concentration, is also required for pollen tube growth and is maintained by the anion efflux transporter S-type anion channel SLOW ANION CHANNEL-ASSOCIATED 3 (SLAH3). CPK2 and CPK20 were reported to enhance pollen tube growth at the pollen tip by activating SLAH3 (Gutermuth et al., 2013). In addition, the *cpk11/24* double mutant showed improved pollen tube growth but impaired Ca^2+^-dependent inhibition of inward K^+^ channels, indicating that these CPKs negatively regulate pollen tube elongation (Zhao et al., 2013). The maize gene *ZmCPK32* is expressed in pollen tube growth; *ZmCPK32* has CPK activity and is localized to the plasma membrane and punctate internal membrane compartments. qPCR and *in situ* hybridization revealed that *ZmCPK32* is expressed at high levels in mature pollen grains, suggesting that it may be functionally linked with pollen tube development (Li et al., 2018). Transient expression of *ZmCPK32* in tobacco repressed pollen tube germination and growth, which depended on its kinase activity. Again, the constitutively active form of *ZmCPK32* reduces seed germination rate, whereas cytosol-localized *ZmCPK32* had a minor effect on pollen tube elongation (Li et al., 2018). These findings support the theory that different CPK members may be linked to different aspects of pollen tube growth.

CDPKs have been found in floral transition, which is initiated by the florigen complex. Florigen is made up of two transcription factors, flowering locus T and D (FT/FD), whose interaction is known to require Ca2+-dependent phosphorylation of FD on T282 by 14-3-3 proteins (Kawamoto et al., 2015b). In a screen of ten nuclear CPKs, *AtCPK33* and *AtCPK6* were identified as the major protein kinases interacting with and phosphorylating FD on T282 *in vitro*. However, single and double *cpk6, 33* mutants showed only a weak delayed in flowering time when compared to the *fd* mutant (Kawamoto et al., 2015b), showing that additional CPKs are likely involved in this process. Transgenic lines expressing a dominant negative variant of *AtCPK33* that can interact with FD without phosphorylating it displayed a distinct late-flowering phenotype (Kawamoto et al., 2015a).

CDPKs are expressed in plant roots, stems, leaves, fruits, and seeds. *IbCDPK28* expression levels increased significantly in sweet potato tuberous roots during tuberization, and may play a functional role in regulating tuberous root growth (Li et al., 2022). The expression of potato CDPK isoforms has been linked to the stolon-to-tuber transition, with *StCDPK1* being involved in gibberellic acid (GA) signaling, indicating a potential role in tuberization (Gargantini et al., 2009). An integrated network centered on the small GTPase ROP (rho of plants) revealed a role for CDPKs in root hair development. The GDP dissociation inhibitor (RhoGDI) and the guanine nucleotide exchange factor (RopGEF) regulate the transition of ROP from GDP-bound inactive to GTP-bound active states (Yip Delormel and Boudsocq, 2019). ROP11 and RopGEF1 impede root hair development, whereas RhoGDI promotes it (Yip Delormel and Boudsocq, 2019). *AtCPK3* has been shown to phosphorylate RhoGDI at three residues: S45/S48/T52 (Wu et al., 2013). Unlike a phosphodead variant, a phosphomimic variant, which had a higher affinity for ROP, could rescue the *gdi* mutant’s defective root hair morphology, demonstrating a positive role of *AtCPK3* by stimulating RhoGDI. Table 1 provides an overview of some identified CDPK genes in plants and their known biological functions.

**TABLE 1:** CDPKs and their biological functions.

Gene	Organism	Sub localization	Gene function	References
AtCPK32	Arabidopsis thaliana	Expressed in protoplast	Enhances ammonium uptake regulation in root	Qin et al. (2020)
AtCPK33	Arabidopsis thaliana	Not identified	Induce stomatal closure	Wang et al. (2019)
ZmCPK32	Zea mays	Plasma membrane	Regulate pollen tube growth and germination	Li et al. (2018)
PnCDPK1	Pharbitis nil	Cytosol	Regulate flower development	Jaworski et al. (2012)
HbCPK9/15	Hevea brasiliensis	Cytoplasm	Implicated in ethylene-simulated latex production, induced abiotic stresses	Xiao et al. (2017)
BdCDPK2/3/8	Brachypodium distachyon	Cytoplasm, chloroplast, mitochondrial	Involved in phytohormones signaling	Wen et al. (2020)

### 5.4 Role of CDPKs in biotic and abiotic stress response

The major biological function of CDPKs is associated with plant response to abiotic and biotic stresses. Differential expression of CDPKs has been found in response to a variety of stimuli, including ABA, drought, salinity, cold, pathogens, and wounding (Ray et al., 2007; Romeis et al., 2001; Wan et al., 2007). In rice, stress-responsive cis-elements have been identified as the promoters of its CDPK genes (Wan et al., 2007). Table 2 shows some known CDPKs involved in signal transduction pathways leading to various stress responses.

**TABLE 2:** Physiological role of CDPKs in plants and their subcellular location under biotic and abiotic stresses.

Gene	Organism	Treatment (conc./method)	Reagent/organism	Sub localization	Biotic and abiotic stress response	References
OsCPK24	Oryza sativa	4°C	Cold	Cytosol	Enhance cold tolerance	Liu et al. (2018)
OsCPK17	Oryza sativa	4/5°C	Cold	Golgi or Trans-Golgi network (TGN)	Enhance cold tolerance	Almadanim et al. (2017)
OsCPK21	Oryza sativa	200 mM, 1 µM	NaCl, ABA	Plasma membrane, cytoplasm	Modulate abscisic acid and salt stress responses, promote growth and development	Chen et al. (2017)
OsCPK4	Oryza sativa	100 mM, 20% PEG	NaCl, PEG 8000	Plasma membrane	Enhance salt an d drought stress tolerance	Campo et al. (2014)
ZmCPK1	Zea mays	4 °C	Cold	Expressed in mesophyll protoplasts	Negatively regulate cold stress signaling	Weckwerth et al. (2015)
CgCDPK	Chenopodium glaucum	300 mM, 20% PEG	NaCl, PEG 6000	Plasma membrane	Respond positively to salt and drought stress	Wang J. et al., (2017)
ClCDPK14	Citrullus lanatus	300 mM, 4 °C, unwatered	NaCl, cold, drought	Not identified	Promote cold, drought, and salt tolerance	Wei et al. (2019)
GhCPK55/96	Gossypium hirsutum	400 mM	NaCl	Plasma membrane	Salt stress tolerance	Gao et al., (2018)
FaCDPK1/3/4	Fragaria ananassa	80 mM, 200 µM, unwatered	NaCl, ABA, drought	Chloroplast, nucleus, and cytoplasm	Trigger tolerance to salt, drought, ABA stress. Promote fruit development and ripening	Crizel et al. (2020)
StCDPK3/23	Solanum tuberosum	200 mM	Mannitol	Plasma membrane	Induce photosensitivity and responsive to drought and hormone stimuli	Bi et al. (2021)
VpCDPK6/9	Vitis pseudoreticulata	300 mM, 4 °C, 42 °C, inoculation	Salt, cold, high temperature, *Erysiphe necator*	Plasma membrane, nucleus, cytosol	Immune stress response, salt, cold, high-temperature regulation	(Zhang et al., 2015); (Bredow et al., 2020); (Matschi et al., 2015); (Monaghan et al., 2014)
TaCPK4	Triticum aestivum	200 mM, 4 °C, 5 µM	NaCl, cold, GA	Cytosol and the nucleus	Respond to salt, cold, ABA and GA stress, pathogen defense	Li et al. (2008)
StCDPK7	Solanum tuberosum	25 sporangia/µl	*Phytophthora* zoospores	Cytosolic or nuclear	Confers pathogen resistance	Fantino et al. (2017)
AtCPK3/13	Arabidopsis thaliana	Second-and third-instar larvae	*Spodoptera littoralis*	Nuclear, cytosolic, and plasma membrane	Defense response against insect herbivory invasion	(Dammann et al., 2003); (Kanchiswamy et al., 2010)
AtCPK1	Arabidopsis thaliana	10^6^ spores/ml, 10^5^ cfu/ml	*F. oxysporum, B. cinerea, Pto DC3000*	Lipid bodies and peroxisomes	Confers pathogen resistance	Coca and San Segundo, (2010)
AtCPK5	Arabidopsis thaliana	10^4^ cfu/ml	*Pto*DC3000	Expressed in mesophyll protoplasts	Pathogen defense	Seybold et al. (2019)
AtCPK28	Arabidopsis thaliana	100 µM, larvae feeding	CaCl_2_, *Spodoptera littoralis*	Plasma membrane	Involve in defense signaling, contributes to plant growth and development	(Bredow et al., 2020); (Matschi et al., 2015); (Monaghan et al., 2014)

cfu, Colony-forming unit; PEG, polyethylene glycol; *PtoDC3000*, *P. syringae* pv. Tomato; GA, Gibberellic acid; ABAAbscisic acid.

#### 5.4.1 Abscisic acid (ABA) stress response

ABA acts as a chemical signal in response to environmental stresses, triggering the activation of numerous physiological and developmental processes in plants adaptation to stress conditions (Finkelstein et al., 2002; Ton et al., 2009). Several studies have recently described a group of CDPKs as parts of the ABA signaling pathway controlling plant responses to abiotic stresses. For example, *AtCPK32* binds to and phosphorylates the ABA-responsive transcription factor ABF4, and *AtCPK32*-overexpressing plants showed a hypersensitive phenotype to ABA (Choi et al., 2005). As mentioned above, *AtCPK23*, together with *AtCPK21*, regulates the anion channel SLAC1 and SLAH3 to control ABA-mediated stomatal movement (Geiger et al., 2010), where the ABA- receptor RCAR1-ABI1 pathway regulates *AtCPK21* expression (Geiger et al., 2011). Moreover, *AtCPK3* and *AtCPK6* are both positive regulators of ABA signaling in stomatal movement (Mehlmer et al., 2010; Xu et al., 2010), while *AtCPK6* plays a positive role in regulating methyl jasmonate signaling in guard cells resulting in stomatal closure (Ye et al., 2013). The *Arabidopsis* mutants lacking *AtCPK10* expression were impaired in their ability to inhibit ABA-induced stomatal opening (Zou et al., 2010). Additionally, *AtCPK12* interacts with, phosphorylates, and activates the type 2C protein phosphatase ABI2 and two ABA-dependent transcription factors ABF1 and ABF4 (Zhao et al., 2011). ABF1 and ABF4 are ABA-dependent, simple leucine zipper transcription factors that play a positive role in ABA signal transduction (Choi et al., 2000). However, ABI2 plays a negative role in ABA signal transduction (Cutler et al., 2010). When *AtCPK12* is downregulated, *AtCPK12*-induced stimulation of ABI2 phosphatase activity can be abolished, whereas when *AtCPK12* is upregulated, *AtCPK12*-induced repression of transcription factors ABF1 and ABF4 can be relieved. As a result, ABA signaling can be enhanced in both cases, which may explain why both *AtCPK12* overexpression and RNAi lines exhibit ABA hypersensitive phenotypes (Zhao et al., 2011). Exogenous ABA treatment increased the expression of *BrrCDPK38/42* and *FaCDPK4/11* in *Brassica* and *Fragaria*, respectively (Wang et al., 2017; Crizel et al., 2020). In rice, *OsCPK14* and *OsCPK21* interact with and phosphorylate *OsDi19–4* transcription factor (Wang et al., 2016) and 14–3-3 protein (*OsGF14e*) (Chen et al., 2017), respectively, and positively regulate ABA signaling and other abiotic factors.

#### 5.4.2 Drought stress response

**FIGURE 2:**
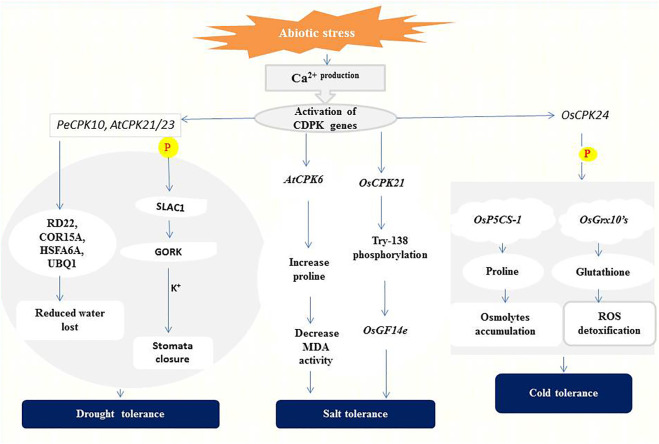
A representation of CDPK functions in abiotic stress signaling. CPK21/23 phosphorylates (**P**) SLAC1 to activate K^+^ ions efflux generation leading to stomatal closure (Geiger et al., 2010). *PeCPK10* on the other hand induces drought tolerance through the expression of drought-responsive genes as stated above (Chen et al., 2013). Overexpression of *AtCPK6* elevates proline level thereby repressing Malondialdehyde (MDA) generation (Xu et al., 2010). Whiles *OsCPK21* interacts with *OsGF14e* by partially phosphorylating Try-138 to initiate the salt tolerance response (Chen et al., 2017). *OsCPK24* phosphorylates (**P**) *OsP5CS-1* to trigger proline production and also interacts with *OsGrx10’s* leading to glutathione generation which detoxifies reactive oxygen species (ROS) (Liu et al., 2018).

Drought is one of several factors affecting crop production and productivity in developing countries (Aravind et al., 2017). The effects of drought stress on plants include reduction in water potential, closure of stomata due to increased accumulation of abscisic acid (ABA), and reduced photosynthesis, biomass, and grain yield (Shikha et al., 2017; Van Gioi et al., 2017). To minimize the adverse effects of drought, the role of CDPKs in regulating plant stress adaptation was investigated to gain more insight into their signaling processes. CPK33 interferes with stomatal closure and slow anion currents (Li et al., 2016). Drought stress and ABA treatment resulted in two unique *cpk33* mutants with significantly smaller stomatal apertures than wild-type plants, as well as drought tolerance and increased slow anion channel activity. CPK33 over-expression lines displayed the reverse phenotypes as mutants, including impaired stomatal closure, increased water loss, and reduced drought tolerance. It was discovered that thiamine thiazole synthase 1 (THI1) physically interacts with CPK33 (Li et al., 2016). THI1 inhibited CPK33 auto-phosphorylation activity in an *in vitro* kinase assay, which is consistent with the phenotypes of THI1 over-expression lines. Similarly, *AtCPK8* has been shown to have positive functions in ABA- and H_2_O_2_-mediated stomatal movement in response to water deficit, leading to stomatal closure (Zou et al., 2015). Under drought stress, *Arabidopsis* CPK23 and CPK21 phosphorylate and activate SLAC1 which leads to membrane depolarization and activation of the K^+^ release channel GORK (Geiger et al., 2011). These changes eventually result in the closure of stomata and reduction or even cessation of transpiration for the adaptation to drought (Geiger et al., 2010) (Figure 2).

The expression of CDPKs in enhancing drought tolerance has been confirmed in several plant species. In ginger (*Zingiber officinale*), its *ZoCDPK1* positively regulates signaling pathways in the response to drought and salt stress in a DRE/CRT-independent manner (Vivek et al., 2013). Overexpression of *ZoCDPK1* in tobacco plants increases the expression level of the stress-related genes ERD1 (EARLY RESPONSIVE TO DEHYDRATION 1) and RD21A (RESPONSIVE TO DEHYDRATION 21A), conferring drought stress tolerance. The rice *OsCPK10* promotes drought tolerance by protecting cell membranes through enhanced ability to remove toxic reactive oxygen species (ROS) (Fang et al., 2015; Yin et al., 2015). *ZmCPK12* is strongly induced by drought in maize seedlings, and overexpression of *ZmCPK12* in *Arabidopsis* improved plant survival during drought (Wang and Song, 2013). Also, *Populus euphratica PeCPK10* improves drought tolerance by activating drought-dependent genes such as RD22, COR15A, UBQ1, etc., and stimulates the expression of numerous ABA-dependent genes (AB11, AB15, NF-YB7, etc.) when overexpressed in *Arabidopsis* (Chen et al., 2013) (Figure 2).

In terms of transcriptional control of CDPKs under drought conditions, cis-elements in their promoters play an essential role. Many *Glycine max GmCDPKs* carry multiple stress-responsible cis-elements; moreover, most paralogs show little concordance in the distribution of cis-elements, suggesting that the promoters of paralogous *GmCDPKs* diverged after genome duplication events (Hettenhausen et al., 2016). Further investigation of the drought-inducible *GmCDPK* genes revealed the presence of ABREs (ABA response elements) and/or MBS (MYB binding sites) in their promoters, and nearly 80% of all *GmCDPKs* responded to either drought, ABA, or both (Hettenhausen et al., 2016). Functional analysis of turnip (*Brassica rapa*) *BrrCDPKs* revealed the up-regulation of many turnip CDPKs under various stress conditions, including drought stress (Wang et al., 2017). In cereals such as foxtail millet (*Setaria italica*), studies of the CDPK gene family revealed 13 and 11 *SiCDPKs* were up-and down-regulated, respectively, under drought, and most *SiCDPKs* were up-regulated by ABA, except *SiCDPK4* and *SiCDPK20* (Yu et al., 2018).

#### 5.4.3 Salt stress response

Soil salinity has emerged as a major threat to global crop production, affecting more than 800 million hectares of cropland, representing over 6% of the world’s land area (Munns and Tester, 2008). Salinity causes osmotic stress and decreases water uptake by plants (Liu et al., 2015). In addition, salinity also causes over-accumulation of Na^+^ and Cl^−^ ions which creates an ionic imbalance in the plant. Plants normally mitigate the lethal effects of elevated Na^+^ in cells through two mechanisms: Na^+^ exclusion and Na^+^ sequestration (Nongpiur et al., 2016). Calcium acts as a second messenger in activating CDPKs gene expression, leading to the synthesis of compatible solutes to stop the deleterious effects of elevated Na^+^ in the cytoplasm (Mahajan et al., 2008). CPK13 prevents stomata opening through its inhibition of guard cell-expressed KAT2 (K^+^ transporter 2) and KAT1 (K^+^ transporter 1) channels (Ronzier et al., 2014). CPK12 on the other hand confers salt tolerance by regulating ion homeostasis and H_2_O_2_ production in roots, whilst downregulation of CPK12 led to high salt sensitivity in seedling growth and accumulation of high levels of Na^+^ and H_2_O_2_ (Zhang et al., 2018). Moreover, Negrão et al. (2011) discovered that *OsCPK17* co-localized with four QTLs that correlate with ion homeostasis and salinity response. In addition, a potential overlap has been found between the signaling networks of salt stress and ethylene in cotton (*Gossypium hirsutum*). *GhCPKs* can be upregulated within an hour by ethephon (ETH) treatment under salt stress (Gao et al., 2018).

Rice has two positive regulators of salt tolerance: *OsCPK7* and *OsCPK12*. *OsCPK7* is mostly expressed in vascular bundles, where water stress is most severe when rice plants are stressed by salt and drought. Transgenic rice overexpressing *OsCPK7* displayed increased salt resistance as well as increased induction of some stress-responsive genes, such as *rab16A* (Saijo et al., 2001). *OsCPK12* promotes salt stress responses by upregulating ROS-scavenging enzymes (*OsAPx2* and *OsAPx8*) and suppressing stress-induced ROS overproduction. *OsCPK12* expression is linked to salt tolerance in rice, with mutations and RNA interference silencing resulting in a decreased tolerance to salt stress (Asano et al., 2012b). In the meantime, overexpression of *OsCPK4* in rice induces the expression of genes involved in lipid metabolism and protection against oxidative stress under salt stress (Campo et al., 2014). According to Xu et al. (2010), high salt concentration is lethal to wild-type *Arabidopsis* plants, but transgenic lines overexpressing *AtCPK6* accumulate more proline and less Malondialdehyde (MDA) which depicts improved stress tolerance (Xu et al., 2010). *OsCPK21* interacts with *Os14-3-3* (*OsGF14e*) to positively take part in ABA signaling and salt stress, partly by phosphorylating Try-138 (Chen et al., 2017) as indicated in (Figure 2). Other CDPKs identified in salt stress regulation in plants species include; *ClCDPK6* in watermelon and its ortholog *CsCDPK14* in cucumber (Xu et al., 2015; Wei et al., 2019) and *VpCDPK9* in grape (Zhang et al., 2015).

#### 5.4.4 Cold stress response

Low temperatures are a major obstacle to the expansion of cultivated areas of some subtropical and tropical crops such as rubber (*Hevea brasiliensis*). Understanding plant response and adaptation would contribute to the development of cold-tolerant crops. The rubber, *HbCPK* (Ca^2+^ sensors) is downregulated after exposure to low temperatures (Xiao et al., 2017). Similarly, low temperatures control the expression of several CDPKs in *A. thaliana*, *O. sativa,* and *P. trichocarpa* (Xiao et al., 2017). In *Brachypodium distachyon*, the expression patterns of *BdCDPKs* vary with different gene members in response to cold and heat stress where several are down-regulated shortly after treatment and four (*BdCDPK16/23/25/30*) being strongly induced, suggesting that some *BdCDPKs* function early, while others respond late (Wen et al., 2020). Cold stress increased or decreased the expression of the *ZmCPK1* and *ZmCPK25* genes in maize. *ZmCPK1* has an inverse relation with the regulation of the cold stress signaling mechanism. Studies on transgenic *Arabidopsis* also revealed that *ZmCPK1* negatively regulates the expression of ethylene response factor (*ZmERF3*) genes and impairs cold stress tolerance (Weckwerth et al., 2015).

Several studies have shown that cold-induced expression of CDPKs could lead to a slowdown of cellular metabolism or mounting protection against cold stress. In rice, Ma et al. (2015) discovered that the perception of low temperature is associated with changes in Ca^2+^ influx and that this transition is regulated by COLD1, a G-protein signaling regulator. The rice *OsCDPK13* and *OsCPK17* are considered essential signaling components in cold response (Komatsu et al., 2007; Almadanim et al., 2017), and *OsCPK17* relays cold stress signals and negatively regulates overall metabolism by reducing the activity of enzymes involved in sugar and nitrogen metabolism (Almadanim et al., 2017). On the other hand, overexpression of *OsCPK24* in rice significantly increased proline and glutathione contents during cold treatment likely through up-regulating proline synthetase gene *OsP5CS-1* and promoting Ca^2+^-responsive thioltransferase activity of *OsGrx10* (Liu et al., 2018), as shown in (Figure 2). Both proline and glutathione are well-known protectants against stress because proline contributes to cellular osmotic changes while glutathione helps detoxify ROS (Verbruggen and Hermans, 2008; Hayat et al., 2012). *PeCPK10* in *Populus euphratica* (Chen et al., 2013) and *VaCPK20* in *Vitis amurensis* (Dubrovina et al., 2015) have also been described as positive regulators of cold stress tolerance.

#### 5.4.5 Defense against pathogens/diseases

**FIGURE 3:**
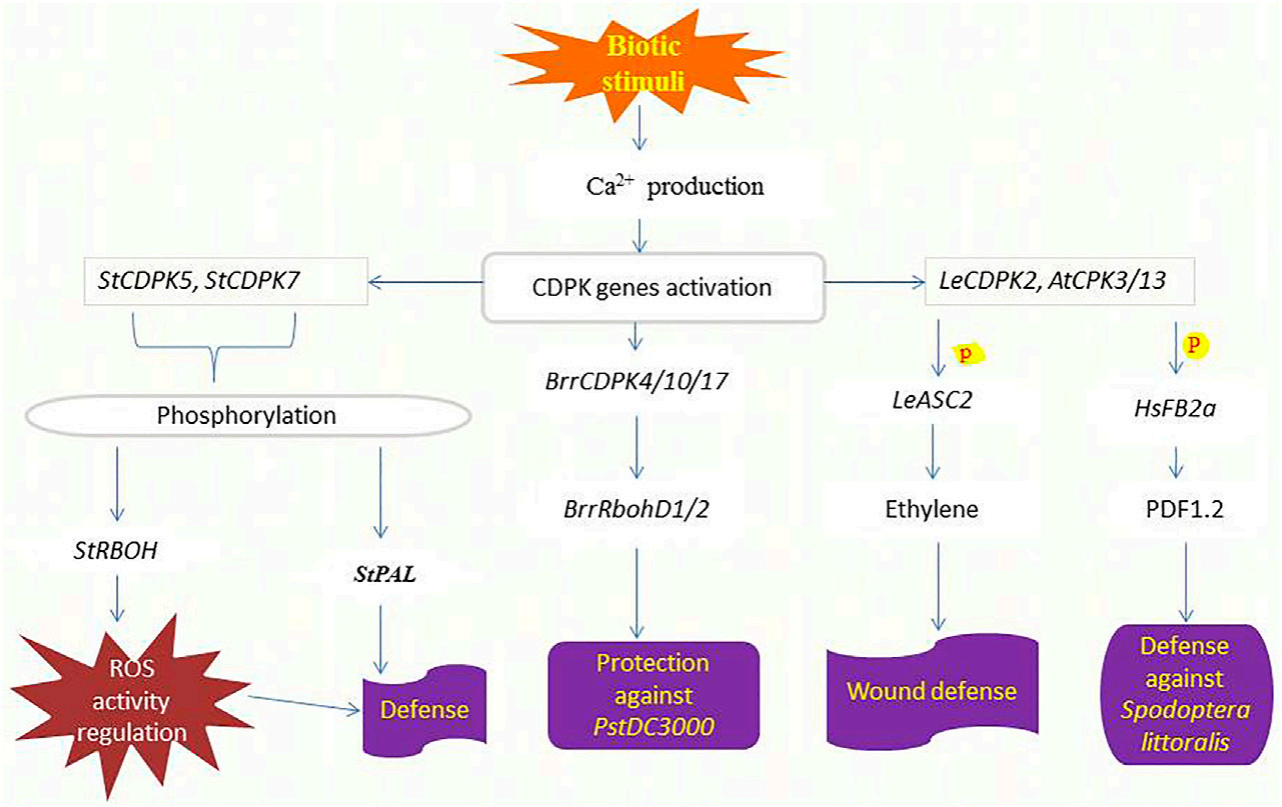
Functions of CDPKs in defending crops against pathogens and herbivore attacks. Upon sensing biotic stimuli or danger, the production of Ca^2+^ increases leading to the activation of various CDPKs responsible for inducing pathogen or herbivory resistance. *StCDPK5* phosphorylates *StRBOH* to regulate ROS activity hence conferring defense response (Kobayashi et al., 2007), likewise, *StCDPK7* phosphorylates *StPAL* to induce defense against *Phytophthora infestans* (Fantino et al., 2017). *BrrCDPKs* interact with *BrrRbohD1/2* to repress the reproduction of *pstDC3000* (Wang et al., 2017). Elevated cytosolic Ca^2+^ triggers the production of *LeCDPK2* upon wounding which phosphorylates (**P**) *LeASC2* leading to the generation and accumulation of ethylene and finally the induction of defense-related genes for protection (Kamiyoshihara et al., 2010). *AtCPK3/13* also responds to wounding by phosphorylating (**P**) *HsFB2a* thereby regulating the transcript level of the PDF1.2 to induce resistance against *Spodoptera littoralis* attacks (Kanchiswamy et al., 2010).

Plants have evolved a multilayered inducible immune system that ensures their survival when attacked by microbial pathogens, including recognition and response to the threat or danger. Intracellular immune responses are rapidly mediated by receptor-mediated perceptions, manifested by adjustments in ion fluxes across membranes, transcriptional reprogramming, and activation of phosphorylation cascades (Romeis and Herde, 2014), where CDPKs and one of their substrates Rboh are involved (Kobayashi et al., 2012; Yoshioka et al., 2016). Wang et al. (2017) discovered that *Pseudomonas syringae* treatment in tomato (*pstDC3000*) increased the expression of most CDPKs. Phytohormones such as ET, JA, and SA with functional roles in biotic stress response can trigger the expression of some *BrrCDPKs*, and the expression of *BrrCDPK4/10/17* was two times higher by *pstDC3000* infection (Dempsey et al., 1999; van Loon et al., 2006). Both hormones and *pstDC3000* infection also upregulate *BrrRbohD1/2* expression (Figure 3), indicating the involvement and the interaction between *BrrCDPK4/10/17* and *BrrRbohD1/D2* in plant resistance against *pstDC3000* (Wang et al., 2017).

The potato and tobacco orthologs of *StCDPK7* and *NtCDPK2* play functional roles in plant defense responses triggered by fungal elicitors (Romeis et al., 2001; Coca and San Segundo, 2010). Other CDPKs involved in the plant response to *Phytophthora infestans* in potato, probably target different proteins depending on their subcellular localization. *StCDPK5* is localized in the plasma membrane and phosphorylates *StRBOH* regulating ROS activity (Kobayashi et al., 2007). Overexpression of *StCDPK5* in potato results in tolerance to the hemibiotrophic pathogen *P. infestans* but enhances susceptibility to the necrotrophic pathogen *Alternaria solani* (Kobayashi et al., 2012). Besides that, *StCDPK7* phosphorylates *StPAL1 in vitro*. Both proteins are cytosolically localized and up-regulated in response to *P. infestans* infection, and phosphorylation of *StPAL1* mediated by *StCDPK7* may affect PAL activity and localization associated with defense response (Fantino et al., 2017). Figure 3 summarizes the function of CDPKs in plant defense against pathogenic diseases. Future research will be required to determine whether the different CDPKs communicate with each other to control the projection of defense signals and plant resistance.

#### 5.4.6 Wounding stress and protection against herbivory attacks

After herbivore feeding, there is a significant Ca^2+^ influx restricted to some cell layers in the damaged zone (Maffei et al., 2007). Phosphorylation cascade mediated by mitogen-activated protein kinases (MAP) and JA pathway is used to mediate these initial signals from insect attack within the plant (Howe and Jander, 2008). Kanchiswamy et al. (2010) identified the involvement of two *Arabidopsis* CPKs (CPK3 and CPK13) in the herbivory-induced signaling network through heat shock factor (*HsfB2a*)-mediated regulation of the defense-related transcriptional pathway. Mutant plants of *cpk3* and *cpk13* exposed to insect attack (*Spodoptera littoralis*) had lower transcript levels of PDF1.2 compared with wild-type plants, as shown in (Figure 3). It is shown that *AtCPK3* can be activated by *flg22* in protoplasts (Mehlmer et al., 2010) and induces the expression of the *flg22-*responsive gene NHL10 (Boudsocq et al., 2010). The activation of defense responses mediated by *AtCPK3* and *AtCPK13* is independent of phytohormone signaling pathways (Kanchiswamy et al., 2010). In comparison, *LeCDPK2* in tomato phosphorylates the ethylene biosynthetic enzyme *LeACS2* at the same site, that is, phosphorylated *in vivo* after wounding, indicating that *LeCDPK2* plays a role in ethylene production in response to wounding (Kamiyoshihara et al., 2010) (Figure 3). Wounding also activates extracellular alkalinization by interfering with plasma membrane H^+^ -ATPase, which in tomato is mediated by membrane-anchored *LeCPK1* (Rutschmann et al., 2002; Boudsocq and Sheen, 2013).

However, the first confirmation that CDPKs affect herbivore performance in plants was recently obtained in *Nicotiana attenuate*, where knockdown of *NaCDPK4* and *NaCDPK5* by virus-induced gene silencing (VIGS) increased JA and JA-Ile levels and distorted formation of secondary protective metabolites, leading to improved immunity to feeding by the specialized insect *Manduca sexta* (Yang et al., 2012; Hettenhausen et al., 2013). Interestingly, *NaCDPK4/5* silenced plants also showed shortened stem elongation (Heinrich et al., 2013), which is identical to the *cpk28 Arabidopsis* single mutant (the closest homologs of *AtCPK28* in *N. attenuate* are *NaCDPK4* and *NaCDPK5*). Reduced stem elongation in *cpk28* mutants is characterized by increased secondary growth, altered vascular architecture, and lignification (Matschi et al., 2013), suggesting that *AtCPK28* activity may strongly influence developmental processes rather than specific defense-related phytohormones signaling pathways (Schulz et al., 2013). *AtCPK28* explicitly phosphorylate BOTRYTIS-INDUCED KINASE1 (BIK1), a kinase required for PAMP-induced ignition of defense signaling (Matschi et al., 2015).

## 6 Negative signaling behavior of the CDPK gene family in some crops

Several CDPKs have been shown to play deleterious roles in plant protection. *HvCDPK3* promotes powdery mildew fungus invasion of barley (*Hordeum vulgare*) host cells in both compatible and incompatible interactions, while transient expression of constitutively active *HvCDPK4* triggered a cell death response in *N. benthamiana* and *H. vulgare* (Freymark et al., 2007). Overexpression of *OsCPK12* renders rice susceptible to both virulent and avirulent tuber leaf fungi, possibly due to ABA hypersensitivity and a decrease in ROS production (Asano et al., 2012b). When *OsCPK13* is ectopically expressed in sorghum (*Sorghum bicolor*), it results in cell death, PR protein aggregation, and upregulation of certain protective genes (Mall et al., 2011).

## 7 Conclusion

Calcium-dependent protein kinases (CDPKs) in plants have received considerable attention in recent years because of their role in signal transduction in response to adverse conditions. Under stress conditions, various Ca^2+^ signals are generated, which are identified and decoded by specific Ca^2+^ transducers. This triggers various physiological and biochemical responses in the plant. However, the functional specificity of the different CDPK isoforms derives from their ability to recognize Ca^2+^ signals and bind Ca^2+^ with different affinities, interact with different proteins, and bind precise and exclusive substrates at different subcellular sites. This versatility is critical because plants in the field are very often exposed to a range of different biotic and abiotic stresses. Recent research has shown that plant responses to a mixture of different biotic and abiotic stressors are unique and cannot be directly inferred from the study of individual stressors. Such considerations are critical if we are to develop plants that are more resilient to more than one stress in order to reduce the adverse effects of expected global climate change on agricultural productivity worldwide.

Over the years, the focus has been on the study of biotic and abiotic stress tolerance in plants, including pathogens and herbivores defenses, hormonal signaling, drought, salt, and cold resistance, and other plant developmental processes. Despite extensive studies on the expression profiles of CDPK genes; their precise role in regulating adverse stress transduction pathways remains unclear. Considering the importance of CDPK genes in crop production, the developed transgenic plants should be tested in commercial fields to explore their interaction with an open environment. The positive functional role of CDPK genes in higher plants such as trees (rubber tree), legumes (soybean), tubers (potato), cereals (rice, maize), ginger, tomato, and others provide a solid foundation for future research to explore and exploit their full utility in food crops to mitigate the adverse effects of biotic and abiotic stresses. Progress in this area will help minimize the high cost of control measures associated with biotic stress in agriculture, resulting in high economic returns for industry players.
